# Ratings of performance in multisource feedback: comparing performance theories of residents and nurses

**DOI:** 10.1186/s12909-020-02276-1

**Published:** 2020-10-12

**Authors:** Muhammad Tariq, Marjan Govaerts, Azam Afzal, Syed Ahsan Ali, Tabassum Zehra

**Affiliations:** 1 Department for Educational Development & Department of Medicine, Aga Khan University, P.O. Box 3500, Stadium Road, Karachi, 74800 Pakistan; 2 School of Health Professions Education, Maastricht University, Maastricht, Netherlands; 3 Department for Educational Development and Department of Medicine, Aga Khan University, Karachi, Pakistan; 4 Department of Medicine, Aga Khan University, Karachi, Pakistan

**Keywords:** Multisource feedback, 360-degree evaluation, Performance theories, Residents, Nurses, Ratings, Assessors

## Abstract

**Background:**

Multisource feedback (MSF) is increasingly being used to assess trainee performance, with different assessor groups fulfilling a crucial role in utility of assessment data. However, in health professions education, research on assessor behaviors in MSF is limited. When assessing trainee performance in work settings, assessors use multidimensional conceptualizations of what constitutes effective performance, also called personal performance theories, to distinguish between various behaviors and sub competencies., This may not only explain assessor variability in Multi Source Feedback, but also result in differing acceptance (and use) of assessment data for developmental purposes.

The purpose of this study was to explore performance theories of various assessor groups (residents and nurses) when assessing performance of residents.

**Methods:**

A constructivist, inductive qualitative research approach and semi-structured interviews following MSF were used to explore performance theories of 14 nurses and 15 residents in the department of internal medicine at Aga Khan University (AKU).

Inductive thematic content analysis of interview transcripts was used to identify and compare key dimensions in residents’ and nurses’ performance theories used in evaluation of resident performance.

**Results:**

Seven major themes, reflecting key dimensions of assessors’ performance theories, emerged from the qualitative data, namely; communication skills, patient care, accessibility, teamwork skills, responsibility, medical knowledge and professional attitude. There were considerable overlaps, but also meaningful differences in the performance theories of residents and the nurses, especially with respect to accessibility, teamwork and medical knowledge.

**Conclusion:**

Residents’ and nurses’ performance theories for assessing resident performance overlap to some extent, yet also show meaningful differences with respect to the performance dimensions they pay attention to or consider most important. In MSF, different assessor groups may therefore hold different performance theories, depending on their role. Our results further our understanding of assessor source effects in MSF. Implications of our findings are related to implementation of MSF, design of rating scales as well as interpretation and use of MSF data for selection and performance improvement.

## Background

Given complexity of workplace settings and interpersonal relations, evaluation of trainee performance by a single assessor (supervisor) is no longer considered defensible. All individuals who interact with trainees on a regular basis can provide meaningful judgments, each from their own perspective, and aggregation of multiple performance data will then provide a more complete and accurate picture of trainee performance [1]. Therefore, Multisource feedback (MSF) is increasingly being used in the assessment of trainee performance in residency training. MSF is a tool which requires collection of evaluations of training- or job-related competencies from various assessors occupying different roles relative to the trainee [2–5]. In health professions education, assessors in MSF may include faculty, fellow residents, medical students, nurses, ancillary staff, patients, families, and the resident him- or herself. Commonly used rating scales in MSF then require assessors to convert trainee performance into numerical scores as well as provide additional narrative comments. Although MSF data are being used as evidence in high stakes decision making about fitness to practice, a major purpose of MSF also is to provide feedback which can be used for continuous development and performance improvement. In fact, MSF has emerged as one of the dominant methods to assess professional attitudes and behavior in the workplace and it can be an effective tool for providing formative feedback to the trainees, especially with respect to ‘generic’ or ‘soft’ competencies such as communication, interpersonal skills, teamwork and professionalism [6].

Extensive literature, both in healthcare and in industry, shows that MSF can be practical, and reliable. For instance, Joshi, Ling, & Jaeger, found that a MSF instrument consisting of a 10 item questionnaire filled in by staff nurses, faculty members, allied health professional staff, medical students, patients, fellow residents, and a self-evaluation by residents, yielded reliable evaluations of residents’ competency in interpersonal and communication skills, and could effectively be used to guide formative feedback [7].

However, although studies suggest that MSF is a valuable tool in assessment of trainee performance, the literature also shows that it may be psychometrically challenging, as performance ratings in MSF may suffer from common assessor errors such as halo and leniency [8–10] as well as low (to moderate at best) agreement on ratings across assessor sources (i.e. groups of assessors differing in professional background and role relative to the trainee) [11, 12]. A study by Tariq et al. [13] for instance, showed that mean performance ratings by nurses were significantly lower than ratings from faculty and clinical staff. Similarly, a study by Chandler et al. revealed higher ratings of residents by the nurses and the faculty as compared to patients and families [1], whereas a study by NG et al. [14], demonstrated that assessors who are subordinates are more lenient and are more likely to demonstrate halo effects compared to peers and supervisors. Various explanations may underlie these (partly contradictory) findings with respect to these assessor “source” effects (i.e. between- assessor group differences in MSF) [12]. Previous research findings, for example, suggest that between-assessor differences may result from assessors observing different behaviors in differing work-relationships and contexts [15]. An increasing body of research furthermore suggests that assessors are active information processors who select, interpret, and integrate specific performance information for judgment and decision making, and that processing of performance information (and thus assessment outcome) is influenced by their working relationship with the trainee, their understanding of effective performance, their personal goals as well as (professional) experience [16]. These different conceptualizations of effective job performance are defined as (personal) performance theories. As a consequence, different assessors may hold different performance theories, i.e. may vary in their conceptualizations of what constitutes effective performance and the performance dimensions or aspects that are considered most relevant for the job [17]. Different assessor groups may therefore give different weightings to different dimensions of performance [12]. A study by Govaerts and colleagues [18], for instance, showed that assessors, when asked to directly observe and assess trainee performance, not only differed with respect to the performance dimensions used in assessment of task performance (i.e. what assessors actually paid attention to) but also showed between-assessor variations in interpretation and valuing of specific behaviors – reflecting idiosyncratic assessor effects. Similarly, as use of various assessor groups is a key feature of MSF feedback procedures, it may very well be conceived that assessor groups holding different conceptualizations of performance underpin assessor source effects in MSF: different assessor groups may hold different performance theories, resulting in varying performance dimensions underlying MSF ratings and (significant) between-group differences in ratings of trainee performance. Given findings related to assessor variability in MSF the question may therefore be raised which performance dimensions are actually being used by assessors from different sources when filling out MSF rating forms to evaluate trainee performance.

Exploration of performance theories underpinning MSF ratings seems particularly important as in most MSF procedures, the trainee himself/herself is a crucial assessor source, whose self-assessment allows for a “gap analysis” between self-perceived performance and performance evaluations by others. Discrepancies between trainees’ performance theories and performance theories of other assessor groups may be especially relevant in relation to acceptance and use of feedback. In general, research suggests that acceptance of feedback may vary, and acceptance of negative feedback is especially difficult if it doesn’t resonate with self-perceived performance [6, 19–21]. Research findings rather consistently indicate that self-ratings may differ considerably from ratings by others. For instance, three large studies demonstrated that self-ratings tend to be higher than MSF ratings from other assessor sources, particularly in the less highly rated individuals [22–24]. However, in a recent study by Bullock and colleagues [11], mean resident self-assessment scores were significantly lower than those provided by faculty and other assessors, suggesting that findings regarding self-assessments in multi-source evaluations are dependent on context and (educational) culture. Discrepancies between self and others may result from inaccurate or distorted ratings, but one might also hypothesize that trainees hold performance theories that are different from performance theories used by supervisors, or other health care workers. Since self-ratings are vital in MSF, and acceptance of feedback is probably dependent on residents’ beliefs about what constitutes good performance (i.e. their performance theories), it is important to identify performance dimensions and theories as used by residents in MSF, and how they differ from performance theories and dimensions used by other assessors. A better understanding of residents performance theories may thus further our understanding of acceptance (or non-acceptance) of external feedback.

### The present study

The purpose of the present study, therefore, was to explore performance theories as used by different assessors in MSF. More specifically, we aimed to compare residents’ performance theories with performance theories used by other assessor sources. Building on the findings from previous studies [13, 25], which suggest that performance ratings by nurses differed significantly from ratings by staff and clinicians, we particularly wanted to explore performance theories held by nurses, being vital stakeholders in residency training. Nurses are a very important part of the healthcare teams and they have unique opportunities to observe the day-to-day behaviors of the residents, particularly in the after-hours, during performance of procedures, and in managing emergencies.

## Methods

### Study design

We used a constructivist, inductive qualitative research approach to gain a more in-depth understanding of residents’ and nurses’ performance theories. Semi-structured interviews were conducted to explore performance dimensions that residents and nurses use when evaluating different levels of resident performance. We used one-on-one semi-structured interviews to create a safe environment for participants to elaborate on their assessment beliefs and approaches to performance evaluations.

### Context/setting of the study

The study was conducted at the Aga Khan University Hospital, which is a tertiary care hospital, in the context of residency training in internal medicine. The internal medicine residency programme comprises 4 years of training. The residency programme has been using MSF to assess residents’ performance since 2010. The residents are evaluated by multiple assessors, including faculty, nurses, unit receptionists, wards coordinators (managers), and peer. MSF-procedures include self-assessment of performance by residents. A copy of the MSF questionnaire used at AKU is presented in annexure 1.

## Participants/subjects

A total of 14 nurses and 15 residents participated in this study. Using purposive sampling strategy, nurses were invited to participate in the study based on their involvement and role in residency training and years of experience in interacting with residents. We included nurses with varying degrees of experience (ranging from 2 to 21 years) and various roles and responsibilities in patient care and residency training (seven registered nurses (RNs), two assistant head nurses, two head nurses, one senior manager nursing & two associate nursing directors; five males and nine females). For the residents, we used stratified purposive sampling to include residents at various levels of training (eight senior residents and seven junior residents).

Residents and nurses were invited to participate via email or by verbal communication; participation was voluntary with no financial compensation. Initially 20 interviews for nurses and residents each were planned. In total we conducted 14 interviews with nurses and 15 interviews with residents. Theoretical saturation was achieved for nurses after 11 and for residents after 12 interviews, after which data analysis revealed no new concepts or themes.

## Reflexivity

The principal investigator (PI) was the director of the postgraduate training programmes in the department of medicine at AKU, overseeing all residency and fellowship programmes in the department. He served as internal medicine residency programme director for ten years and was heavily involved in re-structuring of internal medicine programme. During this period he observed that often there were differences in the rating of the same resident’s performance among different assessor groups (faculty, nurses and residents). This assessment phenomenon led him to explore the differences between the different rater groups. All attempts were made to minimize effects from the PIs preconceptions by including other researchers both in data collection and data analysis. The other three researchers were not actively involved in supervision and performance assessment of residents included in this study and had no prior affiliations with the nursing faculty.

## Data collection

Performance theories of residents and nurses were explored using semi-structured interviews. The interview script / guide (Addendum 2) was developed by the research group based on a previous and similar study by Ginsburg et al. [23] Participants were asked to describe an outstanding, a problematic and an average resident with whom they had worked. Descriptions could be about any aspect of residents’ clinical competence. However the description had to be of an actual resident rather than generalized opinion. Probing questions were used to promote specific description of resident performance behaviors.

Two pilot interviews, one with a nurse and one with a resident, were conducted to test and refine the interview script; these participants were not included in the final study sample. Based on the pilot interviews, only small changes were made to the interview guide.

Each interview lasted for about 30 min. Four researchers were involved in conducting the interviews; each interview was conducted by two interviewers. At the beginning of each interview details of the project along with its implications were discussed with interviewees, and written informed consent was taken after asking for permission to audiotape the interview and explaining procedures for assurance of confidentiality. In order to obtain honest and open responses the researchers made an effort to establish rapport with the participants. Researchers periodically discussed and summarized responses to check their understanding and interpretation of interviewees’ responses.

Interviews were transcribed verbatim by an independent professional medical transcriptionist. To enable a member check, all participants received a summary of their interview for verification a few days after the interview was conducted. There were minimal recommendations for change. After all interviews were transcribed, any identification of the interviewee was removed in order to maintain confidentiality. Each interview transcript was assigned a unique code.

## Data analysis

Inductive content analysis is well-suited for research where few or no previous studies of the phenomenon in question exist. The inductive approach enables researchers to identify key themes in the area of interest by reducing the material to a set of themes or categories.

The analysis of interview transcripts was continued alongside data collection to ensure that the interviews were effectively eliciting the types of anticipated description and to determine when theoretical saturation had been reached.

For nurses and residents separately, we used inductive content analysis [26] of interview transcripts through open coding, to identify key themes / categories which were then abstracted and put together as performance dimensions in a model that was assumed to represent the performance theory held by that particular assessor group.

For each of the assessor groups, two researchers with different professional backgrounds (one internist and one medical educationalist) began by independently coding transcripts for outstanding, average, and underperforming residents. Emerging codes were grouped according to themes that were thought to represent dimensions of performance that assessors’ actually used when describing different levels of trainee performance. Researchers met repeatedly to compare and discuss emergent coding structures, until the coding framework was stable. The final coding framework was considered to represent the aggregate performance theory of that particular assessor group (i.e. the set of dimensions used by that particular assessor group to describe and evaluate performance).

## Ethical considerations

Ethical approval was obtained from the Ethical Review Committee (ERC) of the Aga Khan University.

We took precautions to protect the interests of all participants (residents and nurses). Participation was voluntary and full confidentiality was guaranteed. All participants were informed about research procedures in writing, and permission to audiotape interview sessions was obtained. Data were analysed anonymously.

## Results

**Fig. 1:**
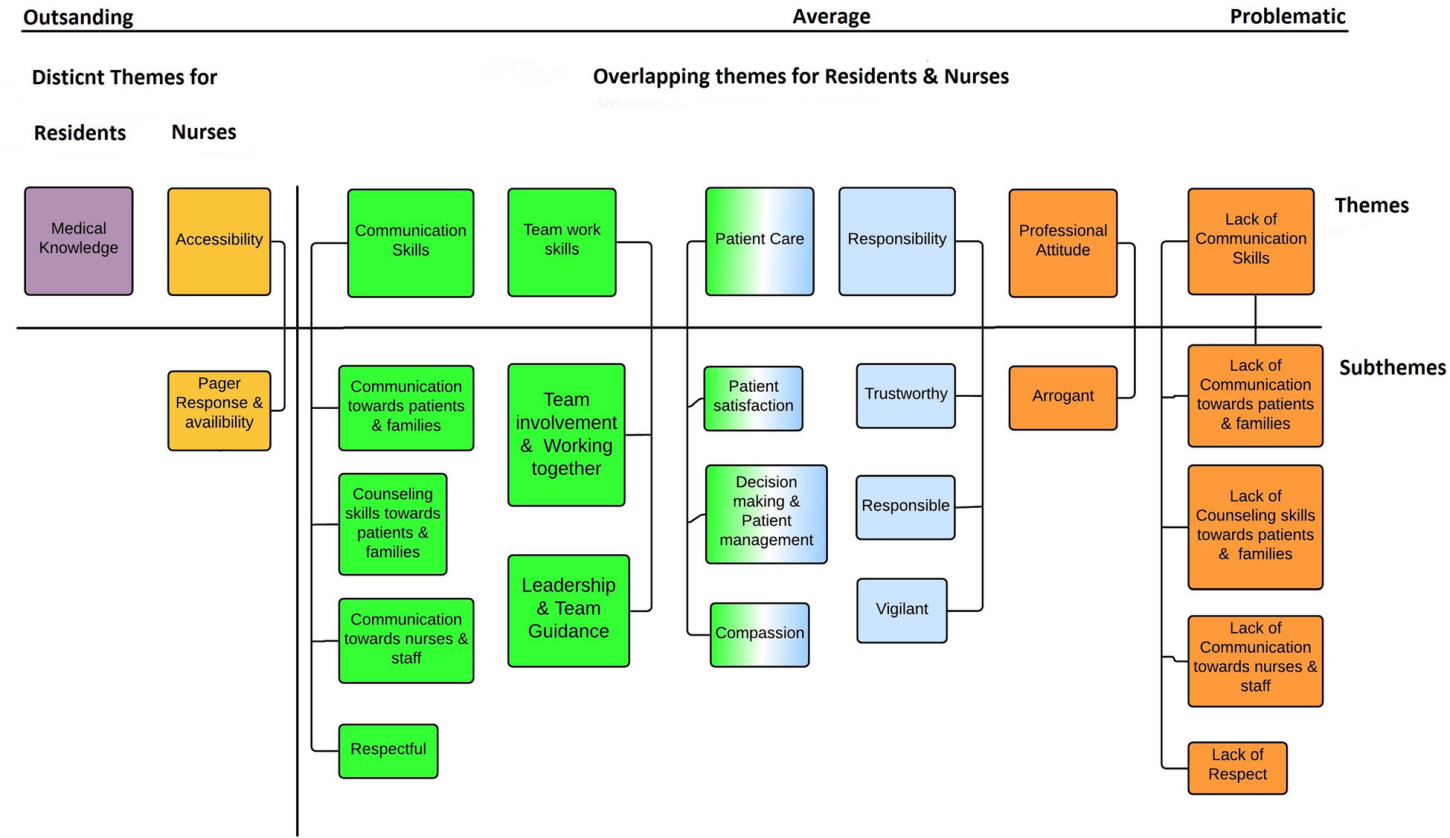
A visual representation of the themes and subthemes derived from residents & nurses responses

The 29 interviews resulted in 184 pages of text for analysis. Analysis showed considerable overlapping, but also meaningful differences in performance theories of residents and the nurses. Fig. 1 shows at a glance the major overlapping and distinct themes and subthemes emerging from our data.

Analysis of the transcripts resulted in seven major themes related to performance theories of nurses and residents, namely; *communication skills, patient care, accessibility, teamwork skills, responsibility, medical knowledge and professional attitude*, The next section will describe these themes in further detail, and provide quotations that most accurately depict the themes and dimensions in participants’ performance theories. Figures indicating participant number are given in parentheses. Table 1 summarizes major findings and provides positive and negative descriptors of residents’ behaviour and performance.

**Table 1:** Performance Theories: Major (sub) Themes derived from interviews, including positive and negative examples of behavioral descriptors

Major themes and subthemes	Definitions	Positive and negative examples of resident behavior / performance	
		Positive descriptors	Negative descriptors
**Communication Skills**			
Communication skills towards patients & families	The act of exchange of information through interaction between residents and patients	Effective communicator Interactive	Ambiguous, rude and bad mannered in verbal and non-verbal communication Intolerant, impatient & aggravated under work stress
Counseling skills towards patients & families	Advice or guidance from a doctor to patients & their families with respect to illness of the patient	Empathetic, maintains confidentiality, confident, explains, listens, available, Educates his/her patients	Poor patient educator and counsellor
Communication skills towards nurses & staff	The act of exchange of information through interaction between residents and nurses	Respect for nurses as healthcare professionals, effective communicator	not available, ineffective direction to nurses, argues, belligerent
Communication skills towards fellow residents (resident)	The act of exchange of information through interaction with residents	Collegial, good interpersonal skills (resident)	aggravated, frustrated & reactive (resident)
**Accessibility**			
Pager response & Availability (nurses)	Response and presence when needed	Prompt response; always available (nurses)	Late response despite multiple pager attempts; not accessible (nurses)
**Patient Care**			
Patient satisfaction	Satisfaction of patients with the healthcare team regarding care of patient	Physician’s presence; timely updates; addresses patient and family concerns Well prepared Grasp on patient issues	upsets and frustrates patient and family Ill informed. Incompetent Poor patient communicator
Decision making & Managing Patients	Treatment & care; Right decision at the right time & competency	confident; competent physician; holistic approach; (nurse) independent assessment. Effective clinical supervision, problem solver, fortitude (resident)	hesitant; lack of confidence; incompetent irresponsible patient care, apprehensive, nervous (resident)
Compassionate (nurse)	Feeling or showing sympathy and concern for others.	Empathetic physician	Insensitive
Safe Practice (resident)	Patient safety taken care of	Gathers thorough information & systemic approach towards patient safety (resident)	negligent, careless casual approach (resident)
**Teamwork Skills**			
Team involvement & Working together	Support & involve the team members in patient care.	collaborative; respect opinions; trust team builder	
Leadership & Team Guidance	Supervision, teaching and managing the team	team leader; mentor; appropriate task delegator	
**Responsibility**			
Trustworthy	Able to be relied on as honest, truthful and dependable	Reliable, credible & dependable authentic (resident)	
Responsible	Having an obligation & commitment for patient care as part of one’s job or role.	responsible & committed	indifferent, unconcerned & uncaring
Vigilant	Keeping careful watch for possible patient deterioration or emergencies	observant, attentive, thorough & cautious	slacker & disinterested
**Professional Attitude**			
Misbehaving	Bad attitude		scolding, stubborn, disrespectful & rude domineering belligerent
**Medical Knowledge** (resident)	Academics, knowledge gain	professional / clinical competence	

### Performance theories, communalities and differences

*Communication* turned out to be the most salient performance dimension in evaluation of resident performance, as this was mentioned and elaborated by all residents and nurses, mostly at the very start of the interview. Both groups indicated that according to them, communication is the key competency or performance domain which differentiates residents as being outstanding, problematic or just average. Particularly good counseling skills make up an excellent resident.

*"Basically what I feel is that the quality that an outstanding resident must possess is communication skills"(Nurse-10, describing outstanding resident performance),*

“*Even the agitated patients used to come around after speaking with him”* “*They felt like they got an answer and felt that the person understands.” (Resident-5, describing qualities of an outstanding resident),*

*"He never discussed in the corridors or at the counter but rather he used to counsel the family in a settled environment". (Nurse-3, describing qualities of an outstanding resident),*

*“Overall with nurses too she communicated very well and still does so.” (Resident-12, describing qualities of an outstanding resident),*

*"Their communication with the family is really pathetic sometimes. Even they say”, “it’s your wish if you want to get treated, this is how it is here".(Nurse-14, describing qualities of a problematic resident),*

The next dominant and common theme was *patient care*. Both nurses and residents emphasized that excellent residents are able to manage patients well with appropriate and high levels of confidence, consistently resulting in high levels of satisfaction in their patients. High-performing residents are compassionate as well as take the right decisions at the right time.

“*In my opinion the outstanding resident is one who goes with the patient treatment plan and focuses on the basic needs of the patient” (Nurse-4; describing qualities of an outstanding resident)*

*“With regard to patient care, he feels no responsibility towards the patient and leaves the management of the patient to the junior doctors from day-1.” (Resident-8; describing qualities of a problematic resident)*

Both residents and nurses also emphasized *teamwork skills*. The subthemes identified within this category were team involvement, working together as partners in patient care, and leadership.

*“An outstanding resident manages a team in a very organized manner”(Resident-3 describing qualities of an outstanding resident )*

*“If the team is fighting then the work will be bad, -----------This will bring quality down.” (Resident-13 describing qualities of an problematic resident)*

*“His behavior with the juniors is like the relationship between a boss and his subordinates.” (Resident-8 describing qualities of a problematic resident)*

Nurses in particular emphasized effective leadership, respect and residents’ ability to make them feel ‘part of the team’. They described an outstanding resident as someone who considers patient care to be a team work effort and nurses to be indispensable team members.

*"He takes everybody along with him" (Nurse-5 describing qualities of an outstanding resident)*

*"She accepts criticism and identifies ways of learning as well as ways of teaching us" (Nurse −1 describing qualities of an outstanding resident)*

*"He was very polite, well communicating when delegating tasks or communicating to his team or to nurses" (Nurse-9 describing qualities of an outstanding resident)*

The sub-theme leadership and team guidance was discussed especially in relation to excellence, whereas the domain seemed to take on less importance in identification of problematic residents, particularly by nurses.

*Accessibility* was identified as major theme by nurses only. Almost all nurses identified this theme when describing resident performance, in contrast to residents who didn’t seem to identify and use accessibility as an important theme in performance evaluations. Nurses typically identified residents as problematic if it is difficult to contact them and if they do not answer their pagers on time.

*"Often we have to page them 3-4 times and the resident does not reply"* (*Nurse-3 describing qualities of a problematic resident*)

*"There are a lot of delays with the incompetent and problematic doctors" (Nurse-4 describing qualities of a problematic resident).*

In distinguishing outstanding residents from residents who are performing poorly, nurses as well as residents highlighted issues related to ‘responsibility’ and ‘professional attitude’. Both residents and nurses used trustworthiness, vigilance, honesty and dependability as key performance dimensions to identify excellence in resident performance. The nurses and residents felt that residents displaying these qualities can easily manage and deal with all sorts of problems.

*“< Outstanding residents> work with responsibility. Their main focus is on responsibility. They focus on the patient’s concerns. They are focused on the treatment plan as well. They follow up on small things such as bed sores management”.* (Nurse 4 *describing qualities of an outstanding resident*).

The participants’ responses clearly identified that problematic residents had issues in professional attitude and poor communication skills. The problematic resident was often described as lacking confidence in patient management and/or not taking responsibility for his patients:

*“Irresponsible with regard to patient care and medication”(Resident 13 describing qualities of a problematic resident)*

*“One who puts responsibility on the nurse and leaves” (Nurse-6 describing qualities of a problematic resident)*

*“Not taking up responsibilities of patients when work is being assigned, and work is then not done” (Resident-2 describing qualities of a problematic resident),*

Problematic residents were also often typified as ‘lacking engagement’, ‘disinterested in their work’, ‘arrogant’ and ‘not committed to patients’ needs’.

Examples of typical quotes were:

“*He has a totally dull and loose posture (Nurse-5 describing qualities of a problematic resident).”*

*“Stubborn, disrespectful, thinks he knows too much and nobody knows anything” (Nurse −14 describing qualities of a problematic resident)*

*“He is rude, thinks that he is superior to us and to other colleagues. No doubt he is knowledgeable but thinks he is superior.”(Resident-7 describing qualities of a problematic resident)*

*Medical knowledge* was not put forward as an important performance dimension in evaluation of residents, neither by residents nor by nurses. The nurses did not discuss it at all, and the residents only referred to medical knowledge when describing outstanding residents.

“*< An outstanding resident> has enough knowledge and is up to date with new research and other things*” (Resident-13 describing *qualities of an outstanding resident*)

Defining performance dimensions for average residents was more difficult for our participants. However the general consensus from participants was that average residents can be thought of as competent (addressing patient care adequately) and responsible yet lacking in communication and teamwork skills. Average residents are actually sort of average, not demonstrating any conspicuous behaviors or competencies that attract attention – either positively or negatively.

## Discussion

In order to gain a better understanding of how assessors and assessor groups evaluate resident performance, we explored and compared performance theories of residents and nurses, as used by them when describing and evaluating different levels of resident performance. Our findings show that performance theories of nurses and residents in our setting are very similar and overlap to a large extent, with only a few differences related to medical knowledge, accessibility and the way they look at team work. Results furthermore showed that performance dimensions may take on varying importance depending on other characteristics of the resident that is being evaluated or discussed. This is in line with the research by Ginsberg et al. [25] which demonstrated that patient communication and leadership were more common in discussions of excellent residents, while trust and residents’ response to feedback were found to be prominent in discussions of problematic residents.

Performance theories that emerged from our data are very much in line with performance dimensions included on the MSF rating form used at AKU, as presented in addendum 1. This may reflect participants’ familiarity with MSF procedures. However, our findings also illustrate that assessor groups attach different weight and meaning to the items on the rating scale; depending on their role and overall judgment of resident performance. Our findings thus seem to illustrate the need to carefully design MSF rating forms to fit various assessor groups, as also suggested by Moonen et.al [27] Our findings furthermore emphasize the importance of narrative comments or narrative evaluations, as nuances and subtle differences between assessor groups may get lost in numeric assessment data.

Communication skills clearly emerge as the most important performance dimension in evaluation of resident performance; it was equally discussed and described by both residents and nurses in outstanding as well as problematic residents. This finding resonates to competency frameworks, which include communication & interpersonal skills as key to high quality patient care [28–31]. Contrary to findings from Ginsburg and colleagues, (where attending physicians were interviewed), our results suggest that non cognitive competencies such as communication and other behavioral competencies are considered far more important than ‘medical knowledge [26]. This may be due to the fact that medical knowledge was not explicitly mentioned in our MSF questionnaire, but an explanation could also be that our study participants (residents and nurses) hold assumptions that every resident has adequate knowledge and skills, with the exception of only a few. In fact, residents did pay attention to medical knowledge, especially when describing outstanding residents. Nurses, however, did not discuss medical knowledge in describing outstanding nor problematic residents, although medical knowledge is considered to be an essential competency [20]. Even when particularly probed, nurses indicated that they do not relate their performance evaluations to medical knowledge of the resident. This is in contrast to the findings by Ginsberg et al., where medical knowledge was found to be one of the major themes [26]. Indeed the nurses in our study strongly believed that it is a resident’s communication skills, professional behavior, team work skills and commitment to patient care that will differentiate him / her amongst peers. . Our findings reflect developments in medical education and health care in general, which emphasize the importance of non-medical expert competencies (communication skills, teamwork skills etc.) in delivery of high quality patient care [25]. Research, however, indicates that most of the feedback in clinical settings is still focused on medical knowledge and clinical skills, neglecting these soft skills, despite stakeholders’ acknowledgement that these competencies may actually differentiate between competent and incompetent trainees [32].

Our findings regarding team work are in line with findings from Ginsburg and colleagues, who identified team interactions and team work to be a major theme in narratives describing outstanding and problematic residents. Our results, however, suggest that residents and nurses look at, and appreciate, teamwork skills in slightly different ways. Residents focus on efficiency in teamwork and task management, whereas nurses focus on respect and acknowledgment of other professionals’ expertise in collaboration in particular. Especially when describing outstanding residents, nurses particularly mentioned residents’ leadership qualities and abilities to “take everybody along”, to guide and support others, to make them feel safe to speak up and part of the team. These findings may reflect very nicely how these groups see each other’s roles in the team, in the hierarchical health care system. As effective team work is thought to be essential in minimizing harmful effects caused by lack of interaction among team members and lack of understanding of individual’s roles and responsibilities [33–36]our findings suggest that team members’ perceptions of their roles responsibilities and expectations in team work need to be made explicit and discussed.

Accessibility in particular emerged as one of the key performance dimensions identified by nurses only. Almost all nurses started by defining a problematic resident as the one who does not answer the pager on time and is not available in the wards at the time of the need. This is understandable as the nurses are very often dependent on residents’ decisions and orders.

The nurses are front line patient care providers along with the residents. The nurse follows the patient care orders written by the residents in the wards. In addition nurses are present all the time in a confined area (wards) but the residents looks after the patients in many clinical areas, including emergency room. As the nurses are dependent on residents’ decisions and orders, it’s very difficult and hard time for them if the resident is not responding and they are in front of the patients. Indeed this is the reason why nurses have categorized non-responders within the problematic category. Accessibility comes under a broader umbrella of professionalism, which is identified as a major competency domain in many competency frameworks [28]. Overall, nurses’ focus on communication, interpersonal skills and professionalism as shown in our study, seems to be in line with findings from the study by Ogunyemi et al. [35], which revealed that residents’ evaluations by the nurses with respect to communication, interpersonal skills as well as professionalism, correlate well with each other but less with faculty (clinicians’) evaluations, which suggests that the evaluations by the nurses offer a unique and important perspective on resident performance that may be useful in performance assessment and formative feedback.

Overall, we feel that our findings suggest that performance theories as held by different assessor groups show commonalities, yet also meaningful differences. This may very well be an explanation for assessor variability in MSF procedures and discrepancies in performance ratings provided by different assessor groups. Findings confirm the need to include various assessor sources in MSF since each assessor group may offer a unique and important perspective on resident performance which may be useful for formative feedback, and may contribute to a holistic picture of a resident’s professional competence [11, 18, 22]. More importantly, however, discrepancies between residents conceptualizations of performance and professional competence and performance theories from others, may result in residents having problems accepting and using feedback for performance improvement, if feedback from assessors does not resonate with their own performance theories.

## Limitations of the study

This study has a number of limitations. MSF traditionally incorporates performance evaluations from many assessor groups, including patients, faculty, medical students etc. In our study, we only focused on performance theories of residents and nurses, and thus may have missed different and complementary views on resident performance. However, we feel that study results further current understanding of assessor source effects. Future studies including other assessor sources may be needed to fully understand relationships between assessor performance theories, assessor variability and source effects in MSF.

Secondly, it was a single center study in an Asian context; therefore, transferability of the results may be questionable.

Finally, participants’ in our study were asked to describe performance of an outstanding, average and problematic resident with whom they had worked. We cannot be sure, however, if and when assessors actually use these dimensions when involved in an actual assessment task. This may be further explored in future studies.

## Implications of our study

First of all, since our findings suggest that assessors may hold different performance theories resulting in assessor variability, future research should explore performance theories of all assessor groups involved in evaluation of resident performance. Future research might furthermore explore relationships between performance theories as held by residents and acceptance and use of feedback in WBA, and MSF in particular. Finally, our findings illustrate the importance of narrative evaluations and put forward the need for further research into development of performance narratives that can be used in design of MSF as well as frame-of reference training for various assessor groups.

Study findings can be used to design MSF questionnaires to adequately reflect performance theories as held by key stakeholders, allowing for differentiated, nuanced and rich portraying of resident performance (and competence levels). Practical implications may furthermore include training of the assessor groups, but more importantly encourage assessor groups to engage in discussion about what constitutes effective performance, and what performance expectations are. The findings this study helped identify areas of improvement and based on this information the MSF form used for our resident assessment was revised.

## Conclusion

Residents’ and nurses’ performance theories for assessing resident performance overlap to some extent, yet also show meaningful differences with respect to the performance dimensions they pay attention to or consider most important. In MSF, different assessor groups may therefore hold different performance theories, depending on their role. When evaluating resident performance, specific differences may exist with respect to valuing of non-cognitive competencies in particular. Study findings may help explain assessor variability in MSF performance ratings.

A better understanding of assessors’ performance theories would help programme directors to develop profiles of outstanding, problematic and average residents, help develop more meaningful assessment tools and criteria and improve the quality of the assessment programme.

## Supplementary information

**Additional file 1.**

## Data Availability

The datasets used and/or analyzed during the current study available from the corresponding author on reasonable request. I can confirm data and material availability.

## Abbreviations

MSF: Multi Source Feedback
AKU: Aga Khan University
RN: Registered Nurse
WBA: Workplace Based Assessment
