# Supplementary material for: Ratings of performance in multisource feedback: comparing performance theories of residents and nurses
Source: BMC Med Educ. 2020 Oct 12;20:355. doi: 10.1186/s12909-020-02276-1 (PMC7549199; doi:10.1186/s12909-020-02276-1)
Supplement: Supplementary file 1 — Additional file 1. [file 12909_2020_2276_MOESM1_ESM.docx]

**Annexure 1 MSF Questionnaire**

**MSF / 360-degree Evaluation of Residents’ Communication and Interpersonal Skills**

Resident being evaluated: Dr. __________________________ Level: R1 R2 R3 R4

Name of Evaluator (optional): __________________________ Gender: M F

Designation: Nurse Head-nurse UR Years of Interaction with Medicine Residents: ____

Faculty Resident Self

Based on the resident’s performance, and on your interactions with the resident or observation of his/her interactions with members of the health care team, patients or their families please rate his/her skills in the following 8 areas. Please rate on a scale of 1 to 7 by marking a tick in the appropriate box, with 1 being poor, 4 being average and 7 being excellent. Any comments that you may have on their performance will also be appreciated.

**Rating 1 – 7 (1 being poor, 4 being average and 7 being excellent). Please mark UC when unable to comment on any particular category.**

| **ITEMS** | **1** | **2** | **3** | **4** | **5** | **6** | **7** | **UC** |  |
| --- | --- | --- | --- | --- | --- | --- | --- | --- | --- |
| **Communication skills and attitude towards staff / nurses** |  |  |  |  |  |  |  |  |  |
| **Teamwork skills** |  |  |  |  |  |  |  |  |  |
| **Compassionate and respectful** |  |  |  |  |  |  |  |  |  |
| **Communication skills and attitude towards patients and their families** |  |  |  |  |  |  |  |  |  |
| **Educating and counseling patients** |  |  |  |  |  |  |  |  |  |
| **Punctuality and accessibility** |  |  |  |  |  |  |  |  |  |
| **Reliability (Dependability)** |  |  |  |  |  |  |  |  |  |
| **Overall professional competence** |  |  |  |  |  |  |  |  |  |
| **Comments (if any):** | | | | | | | | | |

**EXPLANATION GUIDE FOR THE ASSESSORS**

1. **Communication skills and attitude towards staff / nurses**

Please rate how you would describe the resident’s attitude and their manner of communication with nurses, Unit Receptionists and other members of the staff.

1. **Teamwork skills**

How well does the resident interact and work with co-workers (other residents) and how well he/she performs as part of a team.

1. **Compassionate and respectful**

Is the resident kind, courteous and empathetic in his/her interactions with co-workers, staff and seniors?

1. **Communication skills and attitude towards patients and their families**

Does the resident interact with his/her patients and their families in a suitable and respectful manner, showing concern for the patients and families?

1. **Educating and counseling patients**

How well does the resident explain all the relevant information to the patients and their families, and counsels them regarding specific issues?

Eg. A good resident would explain to the patient and their family about the patient’s condition, diagnostic workup, treatment plan, progress, and prognosis in detail, as well as make them a part of all major decisions.

1. **Punctuality and accessibility**

Is the resident regular and punctual for his/her duties, and available easily when required?

1. **Reliability (Dependability)**

Is the resident dependable when entrusted with various responsibilities?

1. **Overall professional competence**

On the whole, how would you rate the resident’s capabilities in performing as a physician and care-giver?

**Annexure 2**

**Interview Script**

Project: **Factors underlying residents’ self-ratings in 360-degree evaluation**

Time of Interview: ------------------------Date: ---------------------Place: --------------------

Interviewer: -------------------------------Interviewee: -----------------------------------------

Position of the Interviewee: --------------------------------------------------------------

Project Details Explained: Yes------No--------

The purpose of the study communicated: Yes------No--------

Confidentiality maintained and re-assured: Yes------No--------

Duration of the interview Communicated: Yes------No--------

Consent form signed: Yes------No--------

Tape recorder turned on and tested: Yes------No--------

**Intended Descriptions:** (There will be more space in the actual form for responses)

1. **Describe a specific outstanding resident with whom you have closely worked?**

-Relevant Probes-------------------------------------------------------------------------

1. **Describe a Problematic resident?**

-Relevant Probes--------------------------------------------------------------------------

1. **Describe an average resident?**

-Relevant Probes--------------------------------------------------------------------------

Thanking the interviewee: Yes------No--------

Assured for confidentiality: Yes------No--------
